# Retirement and elderly health in China: Based on propensity score matching

**DOI:** 10.3389/fpubh.2022.790377

**Published:** 2022-11-03

**Authors:** Xin Peng, Jin Yin, Yi Wang, Xinrui Chen, Liyuan Qing, Yunna Wang, Tong Yang, Dan Deng

**Affiliations:** ^1^Department of Health Statistics, School of Public Health and Management, Chongqing Medical University, Chongqing, China; ^2^Public Health Center, Tianfu New Area Disease Prevention and Control Center, Sichuan, China

**Keywords:** retirement, health, elderly individuals, propensity score matching, genetic matching, generalized boosted model, China

## Abstract

**Background:**

The relationship between retirement and health is important to the formulation of retirement related policies but is a controversial topic, perhaps because selection bias has not been well-addressed in previous studies through traditional analysis methods. Using data from the China Health and Retirement Longitudinal Study (CHARLS), this study explored the potential impact of retirement on the health of elderly Chinese individuals, adjusting for selection bias.

**Methods:**

We balanced the baseline differences between retirement groups and working groups based on nearest neighbor matching and genetic matching with a generalized boosted model (GBM), and regression analysis was used to evaluate the impact of retirement on the health of elderly individuals.

**Results:**

No significant difference was found in any of the covariates between the two groups after matching. Genetic matching performed better than nearest neighbor matching in balancing the covariates. Compared to the working group, the retirement group had a 0.78 (95% CI: 0.65–0.94, *P* = 0.026) times higher probability of self-reported physical pain, a 0.76 (95% CI: 0.62–0.93, *P* = 0.023) times higher probability of depression, and a 0.57-point (95% CI: 0.37–0.78, *P* < 0.001) improvement in cognitive status score. Among male, the retirement group had a 0.89-point (95% CI: 0.45–1.33, *P* < 0.001) improvement in cognitive status score for low education, a 0.65 (95% CI: 0.46–0.92, *P* = 0.042) times higher probability of self-reported physical pain for middle education. For female with low education, the cognitive status of the retirement group was significantly higher by 0.99 points (95% CI: 0.42–1.55, *P* = 0.004), the probability of depression was 0.56 (95% CI: 0.36–0.87, *P* = 0.031) times higher in the retirement group than in the working group. There was no difference for the middle and high education.

**Conclusion:**

Retirement can exert a beneficial effect on the health of elderly individuals. Therefore, the government and relevant departments should consider this potential effect when instituting policies that delay retirement.

## Introduction

As China's medical standards have steadily improved, the overall physical fitness of the Chinese population has also improved significantly, and the average life expectancy has increased in recent years. At the same time, China has long implemented strict family-planning policies to control population size, causing the fertility rate to fall. The extension of life expectancy and the persistence of a low fertility rate have led to a rapid increase in the aging population in China. According to China's National Bureau of Statistics, China has been an aging country since 2000. At the end of 2020, China had two hundred million people aged 65 and above, accounting for 13.5 percent of the population. The aging trend and the labor force shortage have led to the absolute number of China's working-age population and its proportion in the population to decrease annually. Thus, the old-age dependency ratio has increased rapidly and the burden of providing for elderly people has increased.

In China, employees of different genders have different retirement ages. According to the relevant documents issued in 1978 (1, 2), the retirement ages for men and women are 60 and 50, respectively. Men have an older retirement age than women. For employees who have work-related illnesses and are incapacitated, both men and women can retire early at 50 and 45, respectively. Retirement can be delayed for special worker types. Women who are full-time and deputy director-level cadres and professional and technical personnel with senior titles can choose to retire at the age of 60. Most employees should go through retirement procedures at the mandatory age. China's Ministry of Human Resources and Social Security reported that the average monthly pension for enterprise employees in 2020 was ~2,900 yuan (3). According to the data of the National Bureau of Statistics (4), the average annual salary of urban private enterprise employees in 2020 was 57,727 yuan. Retirement implied a partial loss of income capacity. Unlike many developed countries, China has almost no restrictions on whether to work after retirement, and those who have retired can return to the labor market in various forms while receiving pensions.

To alleviate the challenges of caring for the aging population, many countries have put forth initiatives for delaying retirement, aimed at raising the retirement age and prolonging working life through laws and regulations or encouraging policies. For example, France, Germany and Spain will increase the state pension age to 67 years between 2023 and 2029 (5). China also proposed formulating a gradual extension of the retirement age policy. The adjustment of retirement age is related not only to the development of the national economy and people's livelihoods but also to old-age welfare and the quality of life for elderly individuals. At present, most Chinese scholars study whether retirement should be delayed from the perspective of the labor market and the macroeconomic structure and seldom consider it from the health perspective (6–8). However, health is closely related to the quality of life of elderly individuals. For individuals, whether retirement is good for health will affect people's attitudes toward China's policy of delaying retirement. Therefore, exploring the impact of current retirement policies on the health of elderly individuals has scientific importance for the formulation of policies related to progressive retirement age delay.

Scholars in many countries have conducted empirical studies on the impact of retirement on health, but these studies have drawn conflicting conclusions. According to some theories, retirement is a major stressful life transition, carrying negative consequences on health because it entails a loss of benefits intrinsically associated with work, such as social contacts, financial security, a social identity, and structured time (9). On the other hand, activity theory suggests that retirees will have more time to spend with friends or family and engage in a leisurely lifestyle or physical activities, which may have protective effects on health (10). According to the standard stress model of occupational medicine, retirement may represent a disconnect from work-related stressors to improve physical and mental health by reducing work-related risks (11).

In the investigation process, the control of confounding variables, time of investigation and differences in relevant regions may lead to inconsistent results. Yoshinori indicated that for data from the same country, the choice of methods in the research and analysis was the key factor explaining contradictions (12). According to role theory, the retirement choice of elderly individuals is not random and may be related to the burden people bear in the family. For example, people with high life pressures living in poor economies are more inclined to work after retirement to guarantee basic living conditions. Therefore, if confounding factors such as personal basic information, family environment, economic status and social support are not controlled effectively (13–15), it is difficult to truly reflect the relationship between retirement and health. Multivariate regression models, hierarchical analyses, regression discontinuity design (RDD), instrumental variable (IV) and other methods have been adopted by most of the above studies to control for confounding factors. However, different methods have their own limitations in actual data research. For example, a multiple regression model may have multicollinearity (16, 17). Hierarchical analyses cannot address a large number of confounding variables. RDD and IV can only identify local effects and cannot be generalized to the whole population. For example, the estimation results of RDD are only valid in the narrow estimation interval on both sides of the breakpoint. IV exploits some source of exogenous variation, but other factors might simultaneously determine the treatment and the outcome and confound the treatment effect (18).

We adapt the propensity score matching (PSM) method developed by Rosenbaum and Rubin in 1983 to overcome the disadvantages of the above-mentioned methods (19). In this method, the observed covariables are incorporated into the logistic regression model to estimate a propensity value. Then, the intervention group with equal or similar propensity values is matched with the control group to better reduce the selection bias that may affect the results and determine the influence of independent variables. The estimation and matching methods for the propensity values are very important. McCaffrey et al. proposed that a generalized boosted model (GBM) could be used to estimate propensity values, which could improve calculation accuracy (20). Compared with other traditional statistical methods, the GBM has considerable advantages, especially when the functional form cannot be determined by linear, non-linear or interactive relationships between confounding and independent variables (21, 22). The most commonly used matching algorithm is nearest neighbor matching. In 2006, Diamond and Sekhon proposed a new algorithm – genetic matching (23). Its advantages include the uses of a genetic search algorithm to find a suitable weight quickly, which automatically checks and improves the balance of the overall covariates in an iterative way, better achieving balance between the intervention and control groups.

At present, few studies have used PSM analyses to explore the impact of retirement on the health of elderly individuals in China. Most of the available evidence has been obtained through traditional statistical analysis methods (24–27). In this study, cross-sectional data from the China Health and Retirement Longitudinal Study (CHARLS) were analyzed by nearest neighbor matching and genetic matching based on the GBM to balance the covariates between retired and working elderly individuals to better reduce selection bias. The aim was to explore the potential impact of retirement on the health of elderly individuals.

## Methods

### Data

Cross-sectional data were collected from CHARLS in 2018. The CHARLS project is the only large-scale survey conducted by Peking University in China, with middle-aged and elderly people aged 45 and above as the survey objects. It covers multidimensional information, such as health status, physical measurement, family structure, financial support, basic personal information, work, retirement and pensions. The survey randomly sampled households in 450 villages/communities in 150 districts of 28 provinces with a multistage sampling method beginning in 2009 and is regularly followed up every 2 years. CHARLS was approved by the Ethics Review Committee of Peking University, and all participants signed written informed consent (28). CHARLS data can be accessed through the official website (https://charls.pku.edu.cn/en/).

In this study, the missing data of other variables were supplemented and sorted according to the 2015 database. Participants who still had missing values were further excluded. Ultimately, 7,365 individuals were included, including 1,041 in the retirement group and 6,324 in the working group.

### Measurements

#### Independent variable

According to the research purpose, the independent variable was defined as work status (retired or working). The retirement group refers to the group that has already undergone retirement procedures, group excluding inactivity and long-term unemployment, but including disability retirement. The working group refers to those who have not gone through retirement formalities, who have been engaged in economic work in the past year or 1 week, and who were working but may have been on temporary leave, sick leave and other holidays. Individuals who were still working after retirement were considered the working group.

#### Covariates

The covariates in this study included basic personal information, family information, health behavior and social characteristics. Personal basic information included (1) sex, (2) hukou (urban or rural areas), (3) age, (4) education [low level (including uneducated, unfinished primary school, private school, and primary school), middle level (including junior high school, senior high school, and technical secondary school), and high level (including college graduate, bachelor's degree or above)]; and (5) marital status [having a spouse (including married or cohabiting) and others]. Family information included (1) the number of surviving children (none, 1–3, ≥4); (2) economic support [including money and material support, which were obtained by calculating the difference between the financial support received from and given to children in the past year; if the difference was positive (negative), the response was recorded as “received economic support” (“did not receive economic support”)]; and (3) emotional support measured by how often the respondents saw their children. For those who did not live with their children, the frequency of seeing their children ranged from 1 (“almost never”) to 9 (“almost every day”). Those who lived with their children received a maximum of 9 points, and those without children received 0 points. The total score was divided into three groups: 0–2, 3–6, and 7–9 points. Health behavior and disease included (1) smoking (still smoking, given up, never smoked), (2) drinking (more than one drink a month, less than one drink a month, did not drink) and (3) chronic disease (yes, no). Social characteristics included (1) medical insurance (yes, no) and (2) social activities: In the past month, participating in at least one of 11 social activities meant the respondent had social activities; otherwise, the respondent was considered to not participate in social activities.

#### Outcome variables

The outcome variables included self-reported health, self-reported physical pain, life satisfaction, cognitive status, the Activity of Daily Living (ADL) scale, the Instrumental Activity of Daily Living (IADL) scale and depression.

Self-reported health was measured on a 5-point scale ranging from 1 (“excellent health”) to 5 (“poor health”). Life satisfaction was measured on a 5-point scale ranging from 1 (“completely satisfied”) to 5(“not at all satisfied”). Self-reported physical pain was divided into two groups: yes (1) and no (0). Cognitive status was assessed through 11 tests of date cognition, calculation and drawing ability, with a score of 0–11. When the score was higher, the cognitive ability was better.

Self-care ability in daily activities was measured by the ADL scale and the Lawton Functional Scale (29, 30), including the ADL subscale (eating, bathing, dressing, getting in and out of bed, going to the toilet and controlling urination and defecation) and the IADL subscale (managing money, shopping, housework, cooking, and taking medicine correctly). For both the ADL and IADL subscales, each answer was divided into four levels ranging from 0 (“I don't have any difficulty”) to 3 (“I cannot do it”). The scores were summed and defined as the ADL (0–18) and IADL (0–15) scores. When the score was higher, the self-care ability of daily activities was worse.

Depression was assessed by the 10-item Center for Epidemiological Studies Depression Scale (CESD-10). The participants were asked about the number of days they experienced each item in the last week. The total scores ranged from 0 to 30. Participants who had a CESD-10 score of more than 10 were defined as having depressive symptoms, with a code of 1; otherwise, they were defined as symptom-free, with a code of 0. The validity of the CESD-10 has been confirmed among elderly Chinese individuals (31).

#### PSM

As non-randomized studies, observational studies need to reduce the influence of selection bias on the conclusions. PSM can transform multidimensional covariables into a single propensity score (one dimension) and then match two groups of research objects with similar propensity scores so that the covariable distribution of the two groups is balanced and comparable (19).

#### GBM

Logistic regression has been the traditional choice for estimating propensity scores. However, it requires iterative modeling, which can be time consuming and success is not guaranteed, especially if the number of covariables is large. The GBM is a modern multivariate non-parametric regression technique that seeks the optimal propensity score by modeling the logarithmic ratio of intervention selection (20). The formula is


$$\begin{array}{l} g\left(x\right)=\text{logit}\left(\text{e}\left(\text{X}\right)\right)=\log\left(\frac{\text{e}\left(\text{X}\right)}{1-\text{e}\left(\text{X}\right)}\right) \end{array}$$

The GBM has considerable advantages when the functional form of linear, non-linear or interactive relationships between confounding variables and independent variables cannot be determined (21).

#### Nearest neighbor matching

The most commonly used matching algorithm of PSM is nearest neighbor matching. The basic principle is to minimize the difference between the propensity score of participants in the intervention and non-intervention groups and select one participant *i* from the non-intervention group as the match for participant *j* in the intervention group:


$$\begin{array}{l} C\left(P_{i}\right)=\underset{j}{\min}|P_{i}-P_{j}| \end{array}$$

*P*_*i*_ and *P*_*j*_ are the propensity values of the participants in the intervention and non-intervention groups, respectively, and *C*(*P*_*i*_) is the matching set of participants in the intervention and non-intervention groups (32).

#### Genetic matching

Genetic matching, different from the traditional distance matching algorithm, is a generalization of the propensity score and Mahalanobis distance matching; it is a matching method of non-parameter estimation (23). The greatest advantage of genetic matching is the ability to use the genetic search algorithm to quickly find an appropriate weight so the distribution of the covariables involved in the matching can reach the balance between the intervention and non-intervention groups as soon as possible. Genetic matching is based on generalizing the Mahalanobis metric and gives weight parameter w to optimize the matching process. The formula is:


$$\begin{array}{l} GMD\left(X_{i},X_{j},\text{W}\right)=\sqrt{{\left(X_{i}-X_{j}\right)}^{T}{\left(S^{-1/2}\right)}^{T}\text{W}S^{-1/2}\left(X_{i}-X_{j}\right)} \end{array}$$

where *W* is a *k* × *k* positive definite weight matrix; *S* is the variance-covariance matrix of *X*; and *S*^−1/2^ is the Cholesky decomposition of *S*.

### Statistical analysis

In this study, STATA 15.1 was used to convert the CHARLS data, which was in a DTA format into CSV format, and then the data were imported into R 4.0.3 and SPSS 25.0 for the analyses. We preliminarily described the baseline characteristics of the retirement and working groups. Student's *t*-test was used for the continuous variables; the chi-square test or a variance analysis was used for the categorical variables.

This is an observational study, and the influence of selection bias on the conclusion needs to be reduced. Therefore, PSM was adopted to balance the covariables of the retirement and working groups. First, a GBM was used to estimate the propensity score, the shrinkage coefficient was set as 0.0005, and the number of iterations was 25,000. The stop iteration point was set as the average standardized absolute mean difference (ASAM). Second, the treatment case (retired) was matched with the control case (working) by using 1:1 nearest neighbor matching and genetic matching, respectively. The absolute standardized mean difference (ASMD) and ASAM were used to compare the effectiveness of the two matching methods. When the value was smaller, the balance was better. We chose the data matched by better methods for the subsequent analyses.

For the matched data, a linear regression and a logistic regression analysis were used to explore the effect of retirement on outcome variables. For all the results, *P* < 0.05 was used as the standard for statistical significance.

## Results

### Baseline characteristics

Before matching, the baseline characteristics of hukou, age, education level, marital status, number of living children, economic support, emotional support, smoking status, drinking status, chronic disease, medical insurance and social activities of the retirement and working groups were not identical, with the exception of sex; the differences were statistically significant (*P* < 0.05); the maximum ASMD was 1.722, and the ASAM was 0.368 (Table 1).

**Table 1:** Comparison of the baseline characteristics between the two groups before PSM.

**Covariates**	**Retirement group** **(*n* = 1,041), *n* (%)**	**Working group** **(*n* = 6,324), *n* (%)**	***p*-value**	**ASMD**
**Sex**			0.468	0.025
Male	532 (51.1)	3,312 (52.4)		
Female	509 (48.9)	3,012 (47.6)		
**Hukou**			<0.001	1.722
Rural	254 (24.4)	5,632 (89.1)		
Urban	787 (75.6)	692 (10.9)		
**Age**			<0.001	0.804
45–60	135 (13.0)	3,803 (60.1)		
61–75	625 (60.0)	2,390 (37.8)		
76+	281 (27.0)	131 (2.1)		
**Education** ^**a**^			<0.001	0.617
Low level	329 (31.6)	3,803 (60.1)		
Middle level	633 (60.8)	2,390 (37.8)		
High level	79 (7.6)	131 (2.1)		
**Marital status**			<0.001	0.135
Having a spouse^b^	842 (80.9)	5,433 (85.9)		
Others	199 (19.1)	891 (14.1)		
**Number of surviving children**			0.027	0.092
0	16 (1.5)	111 (1.8)		
1–3	873 (83.9)	5,081 (80.3)		
4+	152 (14.6)	1,132 (17.9)		
**Economic support**			<0.001	0.281
Yes	474 (45.4)	3,753 (59.3)		
No	567 (54.6)	2,571 (40.7)		
**Emotional support**			<0.001	0.265
0–2	61 (5.9)	538 (8.5)		
3–6	196 (18.8)	1,791 (28.3)		
7–9	784 (75.3)	3,995 (63.2)		
**Smoking**			<0.001	0.226
Still smoking	224 (21.6)	1,913 (30.2)		
Given up	216 (20.7)	940 (14.9)		
Never smoked	601 (57.7)	3,471 (54.9)		
**Drinking**			0.003	0.111
More than one drink a month	272 (26.1)	1,879 (29.7)		
Less than once a month	118 (11.3)	542 (8.6)		
Did not drink	651 (62.6)	3,903 (61.7)		
**Chronic disease**			<0.001	0.161
Yes	527 (50.6)	2,695 (42.6)		
No	514 (49.4)	3,629 (57.4)		
**Medical insurance**			0.003	0.119
Yes	1,031 (99.0)	6,165 (97.5)		
No	10 (1.0)	159 (2.5)		
**Social activities**			<0.001	0.220
Yes	710 (68.2)	3,646 (57.7)		
No	331 (31.8)	2,678 (42.3)		
**ASAM**	-	-	-	0.368

ASMD, Absolute standardized mean difference; ASAM, Average standardized absolute mean difference.

^a^Low level: uneducated, unfinished primary school, private school, and primary school; Middle level: junior high school, senior high school, and technical secondary school; High level: college graduate, bachelor's degree or above.

^b^Merged with married and cohabiting.

### PSM

**Figure 1:**
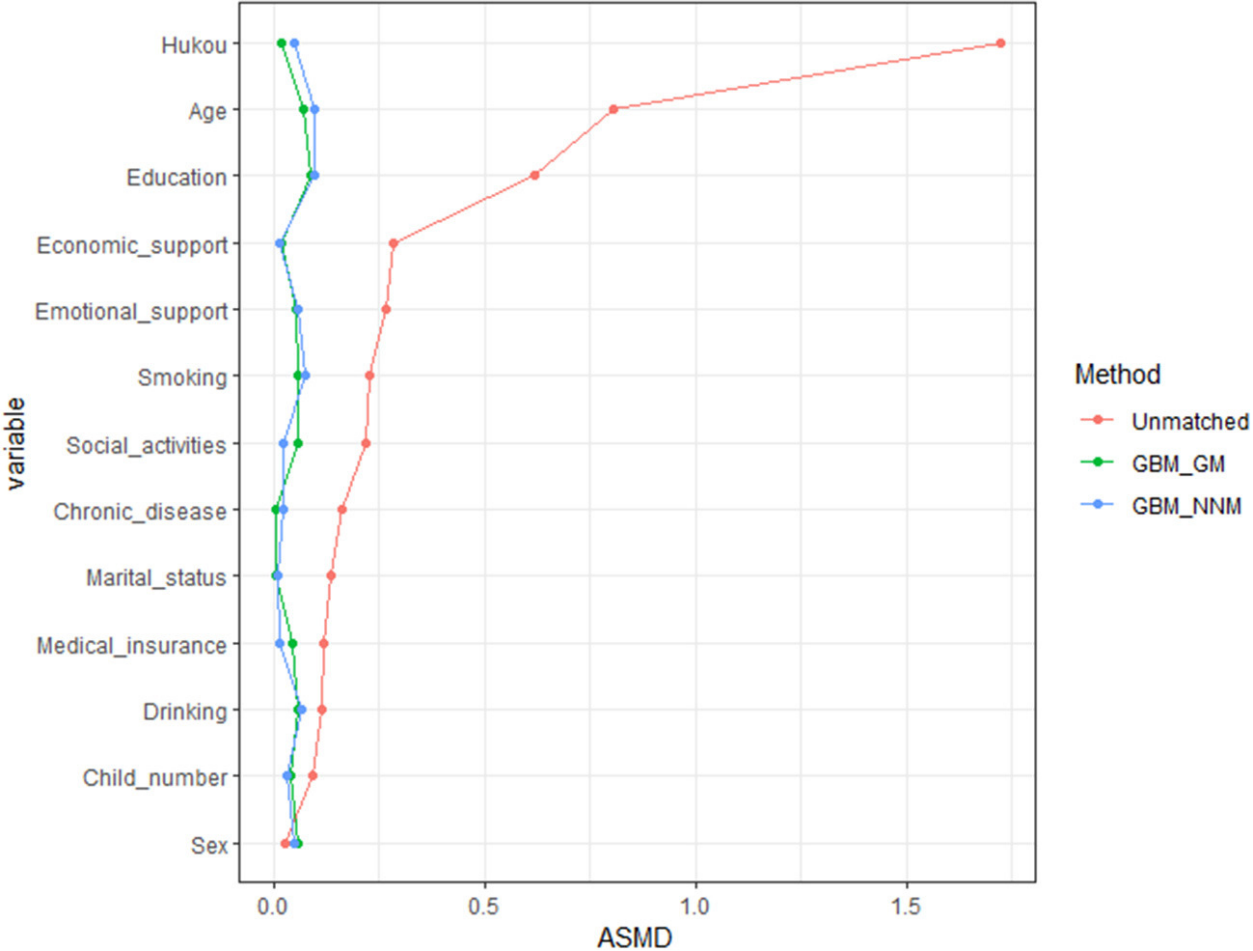
Comparison of the ASMD of the covariates before and after GBM_GM and GBM_NNM (ASMD, average standardized absolute mean difference; GBM_GM, genetic matching based on the generalized boosted model; GBM_NNM, nearest neighbor matching based on the generalized boosted model).

Table 2 and Figure 1 show the baseline characteristics between the two groups after nearest neighbor and genetic matching based on the GBM. In this study, 646 pairs were successfully matched by nearest neighbor and genetic matching based on the GBM. After matching, the balance of the baseline characteristics between the retirement and working groups improved, and the covariates of the two groups exhibited no statistically significant difference (*P* < 0.05). After nearest neighbor matching based on the GBM, the maximum ASMD was 0.097, and the ASAM was 0.046, which were reduced by 94.37 and 87.50% compared to those before matching, respectively. After genetic matching based on the GBM, the maximum ASMD was 0.089, and the ASAM was 0.044, which were reduced by 94.83 and 88.04%, respectively, compared to those before matching. Genetic matching performed better than nearest neighbor matching, so the results of genetic matching were used for the subsequent analysis.

**Table 2:** Comparison of the baseline characteristics between the two groups after nearest neighbor matching and genetic matching based on the GBM.

**Covariates**	**Nearest neighbor matching**				**Genetic matching**			
	**Retirement group** **(*n* = 646),** ***n* (%)**	**Working group** **(*n* = 646),** ***n* (%)**	***p*-value**	**ASMD**	**Retirement group** **(*n* = 646),** ***n* (%)**	**Working group** **(*n* = 646),** ***n* (%)**	***p*-value**	**ASMD**
**Sex**			0.436	0.046			0.344	0.056
Male	344 (53.3)	329 (50.9)			344 (53.3)	326 (50.5)		
Female	302 (46.7)	317 (49.1)			302 (46.7)	320 (49.5)		
**Hukou**			0.421	0.048			0.775	0.019
Rural	250 (38.7)	235 (36.4)			250 (38.7)	244 (37.8)		
Urban	396 (61.3)	411 (63.6)			396 (61.3)	402 (62.2)		
**Age**			0.221	0.097			0.471	0.068
45–60	132 (20.4)	129 (20.0)			132 (20.4)	132 (20.4)		
61–75	378 (58.5)	404 (62.5)			378 (58.5)	395 (61.2)		
76+	136 (21.1)	113 (17.5)			136 (21.1)	119 (18.4)		
**Education** ^**a**^			0.218	0.097			0.280	0.089
Low level	260 (40.2)	262 (40.6)			260 (40.2)	262 (40.6)		
Middle level	350 (54.2)	361 (55.8)			350 (54.2)	360 (55.7)		
High level	36 (5.6)	23 (3.6)			36 (5.6)	24 (3.7)		
**Marital status**			0.940	0.008			1.000	0.004
Having a spouse^b^	540 (83.6)	538 (83.3)			540 (83.6)	539 (83.4)		
Others	106 (16.4)	108 (16.7)			106 (16.4)	107 (16.6)		
**Number of surviving children**			0.874	0.029			0.767	0.041
0	9 (1.4)	8 (1.2)			9 (1.4)	8 (1.2)		
1–3	525 (81.3)	532 (82.4)			525 (81.3)	535 (82.8)		
4+	112 (17.3)	106 (16.4)			112 (17.3)	103 (16.0)		
**Economic support**			0.867	0.012			0.824	0.016
Yes	342 (52.9)	338 (52.3)			342 (52.9)	337 (52.2)		
No	304 (47.1)	308 (47.7)			304 (47.1)	309 (47.8)		
**Emotional support**			0.572	0.059			0.640	0.053
0–2	43 (6.7)	34 (5.3)			43 (6.6)	35 (5.4)		
3–6	140 (21.7)	142 (22.0)			140 (21.7)	144 (22.3)		
7–9	463 (71.6)	470 (72.7)			463 (71.7)	467 (72.3)		
**Smoking**			0.428	0.073			0.595	0.057
Still smoking	152 (23.5)	169 (26.2)			152 (23.5)	160 (24.8)		
Given up	132 (20.4)	118 (18.2)			132 (20.4)	118 (18.2)		
Never smoked	362 (56.1)	359 (55.6)			362 (56.1)	368 (57.0)		
**Drinking**			0.486	0.067			0.585	0.058
More than one drink a month	181 (28.0)	195 (30.2)			181 (28.1)	192 (29.7)		
Less than once a month	72 (11.2)	61 (9.4)			72 (11.1)	62 (9.6)		
Did not drink	393 (60.8)	390 (60.4)			393 (60.8)	392 (60.7)		
**Chronic disease**			0.738	0.022			0.956	0.006
Yes	303 (46.9)	310 (47.0)			303 (46.9)	301 (46.6)		
No	343 (53.1)	336 (53.0)			343 (53.1)	345 (53.4)		
**Medical insurance**			1.000	0.014			0.603	0.043
Yes	637 (98.6)	638 (98.8)			637 (98.6)	640 (99.1)		
No	9 (1.4)	8 (1.2)			9 (1.4)	6 (0.9)		
**Social activities**			0.766	0.020			0.337	0.057
Yes	435 (67.3)	441 (68.3)			435 (67.3)	452 (70.0)		
No	211 (32.7)	205 (31.7)			211 (32.7)	194 (30.0)		
**ASAM**	–	–	–	0.046	–	–	–	0.044

ASMD, Absolute standardized mean difference; ASAM, Average standardized absolute mean difference.

^a^Low level: uneducated, unfinished primary school, private school, and primary school; Middle level: junior high school, senior high school, and technical secondary school; High level: college graduate, bachelor's degree or above.

^b^Merged with married and cohabiting.

### Estimation of the intervention effect of retirement on the health of elderly individuals

As shown in Table 3, after genetic matching based on the GBM, linear regression and logistic regression analyses found that retirement mainly affected self-reported physical pain, cognitive status and depression. Compared to the working group, the retirement group had a 0.78 (95% CI: 0.65–0.94, *P* = 0.026) times higher probability of self-reported physical pain and a 0.76 (95% CI: 0.62–0.93, *P* = 0.023) times higher probability of depression. The cognitive status score of the retirement group was 0.57 points higher than that of the working group (95% CI: 0.37–0.78, *P* < 0.001). In addition, we estimated the health intervention effects of retirement on older individuals of different gender and education levels. For male, the cognitive status score of the retirement group was 0.89 points higher than those of the working group (95% CI: 0.45–1.33, *P* < 0.001) in low education level. The retirement group had a 0.65 (95% CI: 0.46–0.92, *P* = 0.042) times higher probability of self-reported physical pain in middle education level. There is no significant difference in the retirement group and working group on health for the high education level (Table 4). For female with low education level, the results of cognitive status and depression were consistent with those of the whole sample. There is no significant difference in the retirement group and working group on health for the middle education and high education (Table 5).

**Table 3:** Estimation of the intervention effect of retirement on the health of elderly individuals.

**Outcome variables**	**All samples**		
	**β (SE)/OR**	***p*-value**	**95% CI**
Self-reported health	0.03 (0.05)	0.629	(−0.06, 0.12)
Self-reported physical pain	0.78	0.026	(0.65, 0.94)
Life satisfaction	−0.03 (0.04)	0.400	(−0.10, 0.03)
Cognitive status	0.57 (0.12)	<0.001	(0.37, 0.78)
ADL	−0.01 (0.07)	0.907	(−0.12, 0.10)
IADL	0.21 (0.11)	0.054	(−0.004, 0.42)
Depression	0.76	0.023	(0.62, 0.93)

SE, Standard error; OR, Odds ratio; CI, Confidence interval; ADL, Activity of Daily Living; IADL, Instrumental Activity of Daily Living.

Self-reported physical pain and depression were dichotomous variables, and the remaining outcome variables were continuous variables.

**Table 4:** Estimation of the intervention effect of retirement on the health of elderly men individuals of different education levels.

**Outcome variables**	**Male**								
	**Low level**			**Middle level**			**High level**		
	**β (SE)/OR**	***p*-value**	**95% CI**	**β (SE)/OR**	***p*-value**	**95% CI**	**β (SE)/OR**	***p*-value**	**95% CI**
Self-reported health	0.10 (0.12)	0.393	(−0.10, 0.31)	0.02 (0.10)	0.830	(−0.14, 0.18)	0.27 (0.37)	0.470	(−0.35, 0.89)
Self-reported physical pain	0.83	0.435	(0.55, 1.24)	0.65	0.042	(0.46, 0.92)	2.00	0.440	(0.50, 10.46)
Life satisfaction	−0.05 (0.08)	0.509	(−0.18, 0.08)	−0.03 (0.07)	0.641	(−0.16, 0.09)	0.10 (0.20)	0.637	(−0.24, 0.43)
Cognitive status	0.89 (0.27)	<0.001	(0.45, 1.33)	0.34 (0.20)	0.083	(0.02, 0.67)	0.26 (0.49)	0.602	(−0.57, 1.08)
ADL	0.29 (0.18)	0.114	(−0.01, 0.59)	−0.17 (0.09)	0.070	(−0.32, −0.01)	0.10 (0.23)	0.676	(−0.29, 0.48)
IADL	0.50 (0.27)	0.066	(0.05, 0.94)	−0.01 (0.16)	0.932	(−0.27, 0.24)	−0.01 (0.23)	0.979	(−0.39, 0.38)
Depression	0.84	0.509	(0.54, 1.30)	1.03	0.915	(0.67, 1.59)	0.86	0.875	(0.17, 4.91)

SE, Standard error; OR, Odds ratio; CI, Confidence interval; ADL, Activity of Daily Living; IADL, Instrumental Activity of Daily Living.

Self-reported physical pain and depression were dichotomous variables, and the remaining outcome variables were continuous variables.

**Table 5:** Estimation of the intervention effect of retirement on the health of elderly women individuals of different education levels.

**Outcome variables**	**Female**								
	**Low level**			**Middle level**			**High level**		
	**β (SE)/OR**	***p*-value**	**95% CI**	**β (SE)/OR**	***p*-value**	**95% CI**	**β (SE)/OR**	***p*-value**	**95% CI**
Self-reported health	−0.04 (0.14)	0.771	(−0.27, 0.20)	0.01 (0.10)	0.887	(−0.15, 0.17)	0.17 (0.26)	0.523	(−0.28, 0.61)
Self-reported physical pain	0.66	0.105	(0.43, 1.01)	1.10	0.666	(0.76, 1.59)	0.35	0.286	(0.06, 1.66)
Life satisfaction	−0.01 (0.09)	0.903	(−0.16, 0.14)	−0.01 (0.07)	0.909	(−0.13, 0.11)	−0.38 (0.28)	0.185	(−0.87, 0.10)
Cognitive status	0.99 (0.34)	0.004	(0.42, 1.55)	0.25 (0.20)	0.221	(−0.09, 0.58)	−0.82 (0.65)	0.222	(−1.93, 0.30)
ADL	−0.05 (0.21)	0.808	(−0.39, 0.29)	−0.03 (0.09)	0.774	(−0.18, 0.12)	−0.10 (0.10)	0.284	(−0.26, 0.06)
IADL	0.50 (0.27)	0.061	(0.06, 0.94)	0.04 (0.12)	0.737	(−0.15, 0.23)	–	–	–
Depression	0.56	0.031	(0.36, 0.87)	0.67	0.081	(0.46, 0.98)	–	–	–

SE, Standard error; OR, Odds ratio; CI, Confidence interval; ADL, Activity of Daily Living; IADL, Instrumental Activity of Daily Living.

Self-reported physical pain and depression were dichotomous variables, and the remaining outcome variables were continuous variables.

## Discussion

Many countries have raised their retirement ages in the past few years to mitigate the effects of an aging society. The question of whether retirement affects health is important not only in individual retirement decisions but also in the formulation of public policies that influence retirement behavior. Therefore, based on CHARLS data, this study used PSM to explore the possible impact of retirement on the health of elderly individuals to provide a reasonable reference for the formulation of policies related to delaying retirement age.

In view of the imbalance of baseline characteristics between the retirement and working groups, we used nearest neighbor matching and genetic matching based on the GBM to control for the covariates and found that nearest neighbor matching performed worse than did genetic matching, which is a new method for effectively controlling covariates.

The study showed that the elderly people in the retirement group had higher cognitive status scores and lower probabilities of depression and self-reported physical pain than did those in the working group. Disengagement theory provides a possible perspective on the causes of the effects of retirement on the health of elderly individuals. Disengagement theory assumes that social disengagement is an adaptive aging process in which elderly individuals gradually reduce their activity levels and their contact with others and pay attention to their inner life experiences, enabling them to live a calm and satisfying life in their later years and has a positive impact on both society and individuals (33). In addition, the results of this study are consistent with those of other scholars. For example, Lindwall et al. considers that since people's abilities inevitably decline with age, retirees may feel relieved to be free from the pressures of work and competition, and they can pursue their passions and have greater autonomy over their time management (34). Alhainen et al. found that retirees do not have to worry about being late for work, have reduced occupational stress, and get more sleep on weekdays, all of which contribute to mental health (35). Retirement also allows older people to take care of their grandchildren. Eibich proposed that retirement has a positive effect on health through mechanisms such as relieving work-related stress and tension, increasing sleep time, and increasing physical activity. At the same time, caring for grandchildren can improve the subjective health of grandparents, not only alleviating the sense of loss brought about by unemployment, but also improving self-existence value and emotional support (36). In addition, as retirees have more leisure time, the opportunity cost of individual investments in health, such as regular participation in physical exercise, leisure activities and hobbies, is greatly reduced (37). Reduced work stress also frees up time and energy for retirees to strengthen disease prevention and management, which helps reduce physical pain, with positive spillover effects on their mental health and wellbeing (38). Other studies also support our research results, Mein compared people who had retired to those who had worked after retirement and found that retirement improved mental health (39). Research in Australia has shown that retirement has a significant positive impact on women's subjective and objective health indicators (40). At the same time, combined with studies on the health effects of delaying the legal age in some countries, the Netherlands found that delaying the retirement age by 5 years was associated with worsening mental health (41). An Israeli study showed that health deteriorates as retirement age increases (42).

At the same time, we found that for men and women with low education level, their cognitive status after retirement was higher than that of the working group. This may be because people with low education level were often engaged in physical related work. After retirement, they can get rid of heavy and tired work and have more free time to focus on their physical and mental development. Considering the different roles in society between men and women (43), men pay more attention to work, while women have both work and life pressures. After retirement, women are more likely to be influenced by positive social support and neighborhood social cohesion (44), which was good for the development of women's mental health. For women with middle education, men and women with high education, there was no significant difference in outcome indicators between the working group and the retirement group. As scholars have shown, retirement may exert positive health effects on retirees previously employed in more physically and psychologically demanding conditions while a null or even a negative effect is often found among retirees previously employed in good quality jobs (36, 38, 45). This shows that previous work characteristics and their own conditions may be associated with the effect of retirement on health.

The results of this study showed that retirement was generally good for the health of older people. This finding also indicated that the potential impact of retirement on the health of the elderly should be fully considered as China implements a differentiated progressive retirement delay policy. Based on the results of this study, we propose some suggestions: governments could encourage employers and employees to negotiate working hours after employees reach a certain age, in line with changes in labor supply and demand, as this may be beneficial in promoting a win-win situation. In addition, the formulation of retirement age also needs to consider the nature of work, and the qualifications of retirees, such as the official retirement age of manual workers and non-manual workers should be distinguished, so that retirement policies can be further targeted to benefit more people. In addition to physical health, the mental health of elderly individuals needs to be given sufficient attention by society.

There are several limitations to our study that should be mentioned. This study obtained participants' information through questionnaires. The ADL, IADL and CESD-10 scales are self-report-based screening tools rather than clinical diagnostic measures. At the same time, retirement may not only be a determinant of health, but it could be influenced by health itself. We will analyze the relationship between them in follow-up research. The study was a cross sectional design and all variables were measured during the same period. Longitudinal cohort analysis of the relationship between retirement and health should be considered in the future.

## Conclusion

In summary, this study explored the potential impact of retirement on the health of Chinese elderly individuals. PSM was used to minimize the impact of selection bias. The findings showed that genetic matching performed better than nearest neighbor matching based on the GBM in terms of balancing the covariates. This cross-sectional study confirmed the significant association between retirement and health among Chinese elderly individuals. Overall, retirement had a significant protective effect on self-reported physical pain, cognitive status and depression. In addition, there were also differences in self-reported physical pain, cognitive status and depression between the retirement group and working group with different gender and education level. Among men, the cognitive score of the retirement group with low education level was higher than that of the working group, and the probability of self-reported pain of the retirement group with middle education level was lower than that of the working group. For women with low education, the cognitive scores of the retirement group were higher than those of the working group, and the probability of self-reported pain was lower than that of the working group. For women with middle education, men and women with high education, there was no difference in the outcome indicators between the retirement group and the working group. Therefore, the impact on the health of the elderly should be fully considered when formulating policies for progressively delayed retirement.

## Data availability statement

Publicly available datasets were analyzed in this study. This data can be found here: https://charls.pku.edu.cn.

## Ethics statement

The studies involving human participants were reviewed and approved by the Ethics Review Committee of Peking University. The patients/participants provided their written informed consent to participate in this study.

## Author contributions

XP, JY, YiW, and DD: conceptualization. XP, JY, YiW, and YuW: methodology. XP, JY, YiW, and LQ: formal analysis. XP, JY, XC, and TY: writing—original draft preparation. XP, JY, and YiW: writing—review and editing. DD: supervision. All authors have read and agreed to the published version of the manuscript.

## Funding

This study was funded by Humanities and Social Science Research Project of Chongqing Education Commission (Number: 21SKGH028).

## Conflict of interest

The authors declare that the research was conducted in the absence of any commercial or financial relationships that could be construed as a potential conflict of interest.

## Publisher's note

All claims expressed in this article are solely those of the authors and do not necessarily represent those of their affiliated organizations, or those of the publisher, the editors and the reviewers. Any product that may be evaluated in this article, or claim that may be made by its manufacturer, is not guaranteed or endorsed by the publisher.
